# Using chemical biology to assess and modulate mitochondria: progress and challenges

**DOI:** 10.1098/rsfs.2016.0151

**Published:** 2017-04-06

**Authors:** Angela Logan, Michael P. Murphy

**Affiliations:** MRC Mitochondrial Biology Unit, Hills Road, Cambridge CB2 0XY, UK

**Keywords:** mitochondria, targeting, probes, reactive oxygen species, lipophilic cations

## Abstract

Our understanding of the role of mitochondria in biomedical sciences has expanded considerably over the past decade. In addition to their well-known metabolic roles, mitochondrial are also central to signalling for various processes through the generation of signals such as ROS and metabolites that affect cellular homeostasis, as well as other processes such as cell death and inflammation. Thus, mitochondrial function and dysfunction are central to the health and fate of the cell. Consequently, there is considerable interest in better understanding and assessing the many roles of mitochondria. Furthermore, there is also a growing realization that mitochondrial are a promising drug target in a wide range of pathologies. The application of interdisciplinary approaches at the interface between chemistry and biology are opening up new opportunities to understand mitochondrial function and in assessing the role of the organelle in biology. This work and the experience thus gained are leading to the development of new classes of therapies. Here, we overview the progress that has been made to date on exploring the chemical biology of the organelle and then focus on future challenges and opportunities that face this rapidly developing field.

## 1. Introduction

The application of chemical biology to the mitochondrion is a rapidly expanding area that offers the prospect of helping us understand the many facets of this fascinating organelle's rich biology. Interdisciplinary interactions between chemists, physicists, biologists and clinicians are facilitating our ability to both assess and intervene in mitochondrial function, for example, through the development of new chemical tools and therapies. Here, we outline the current status of this evolving field, discuss the many challenges that face us at the moment, and suggest potential ways forward.

## 2. Why study mitochondria?

**Figure 1.**
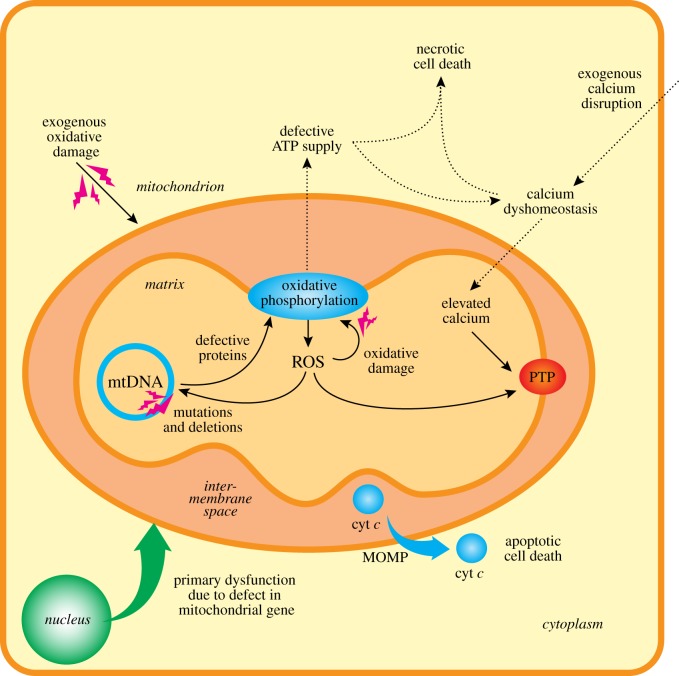
Mitochondrial dysfunction can lead to cell dysfunction and pathology. As is illustrated here, disruption to mitochondrial function can be caused by mutation to mitochondrial or nuclear genes. In addition, mitochondrial dysfunction can arise due to causes outside the mitochondrion, such as increased oxidative stress, disruption to calcium homeostasis and defective mitochondrial ATP synthesis. Frequently, these occur together or lead into each other. In particular, the combination of elevated mitochondrial matrix calcium and oxidative stress leads to induction of the MPTP, which leads to necrotic cell death. In addition, mitochondria can induce apoptosis by mitochondrial outer membrane permeabilization that leads to the release of factors such as cytochrome c (cyt c) that activates cell death by apoptosis.

In considering why to take an interdisciplinary approach and use chemical biology to investigate mitochondria the first question is why focus on mitochondria in the first place? Our answer is that mitochondria are central to the life and death of the cell [1–3]. It is well known that mitochondria are the site of oxidative phosphorylation; consequently, they are the major source of ATP in most eukaryotic cells making any defects to these processes critical for cell survival [1,2] (figure 1). The metabolic pathways, notably the Krebs cycle and fatty acid oxidation that break down carbohydrates, amino acids and fat so that the electrons can be fed into the respiratory chain are vital for oxidative phosphorylation, but their location within mitochondria and their roles at the heart of intermediary metabolism impact many other processes beyond the supply of ATP [1,2,4,5]. Furthermore, the metabolic roles of mitochondria also include many other biosynthetic processes, such as the assembly of FeS centres and haem biosynthesis [6,7]. An interesting recent illustration of the many roles of mitochondrial metabolism has been the discovery that many intermediates in the Krebs cycle also have regulatory roles in the cytosol, such as in hypoxia sensing and altering epigenetic modifications to the nuclear genome, and that these can be disrupted in some forms of cancer [8–10].

The many roles carried out by the mitochondrion are closely integrated into the function of the cell. For example, the mitochondrion is intimately associated with the endoplasmic reticulum [11], which assists in determining the fission of the organelle, and also the movement of calcium from the ER to the cytosol and from there into the mitochondrial matrix as a way of modulating the activity of matrix dehydrogenases and coupling mitochondrial ATP production to the energy requirements of the cell [12,13]. The coordinated, continual fission and fusion of mitochondria is one of the ways in which the continuous turnover of damaged mitochondria is brought about [11]; however, this repair of mitochondrial damage also occurs via several parallel pathways that occur autonomously via proteases and other enzymes within the organelle [14].

Another facet of the role of the organelle in the cell is that mitochondria are intimately involved in cell death [1,12]. This is because the mitochondrial pathway of apoptosis involves the release of factors such as cytochrome *c* from the intermembrane space as a critical step in committing the cell to activating the apoptotic cell death programme [15]. As well as these key forms of regulated cell death, the central role of mitochondria in ATP production means that damage to the organelle will lead to necrotic cell death, due to the lack of ATP preventing the cell from sustaining ion gradients. During necrotic cell death, the induction of the mitochondrial permeability transition pore (MPTP) is a major player, committing the cell to a rapid death [16–18]. The roles of mitochondria also impact on whole-body physiology. For example, in mammals the leak of protons across the inner membrane via uncoupling protein 1 in brown adipose tissue is a major way in which heat is generated [19]. In fact, it is likely that proton leak through the mitochondrial inner membrane independently of UCP1 is a major component of the basal metabolic rate, and thus a central component of thermogenesis in poikilotherms [20].

Mitochondria are also a major source of reactive oxygen species (ROS) within the cell [21–23]. These ROS come from the respiratory chain, primarily in the form of superoxide that then goes on to form hydrogen peroxide [21]. These ROS can overwhelm the multitude of antioxidant defences within the mitochondrial matrix and thereby cause extensive oxidative damage to mitochondria, which contributes to a wide range of pathologies [3]. More interestingly, is the growing view that the production of ROS from mitochondria can act as a redox signal to the rest of the cell, suggesting that the production of ROS by mitochondria may be a way in which the mitochondria ‘talk’ to the rest of the cell coordinating the function of the mitochondria with that of the cell [21–24]. One situation in which mitochondrial ROS signalling seems to be particularly important is in the activation of cells such as macrophages during inflammation [25], and also when mitochondria act as signalling hubs in the response to viral infections [26,27]. These are perhaps related to a signalling role for mitochondrial ROS in the dramatic switch from oxidative phosphorylation to aerobic glycolysis, which occurs in several biomedically important areas such as inflammation and most notably in cancer (i.e. Warburg metabolism) [8,28].

So far, there has been an unstated bias towards mammalian/human mitochondria. The reasons for this are obviously that mitochondria are central to so many biomedically important situations. However, it is important to remember that the ‘mammalian’ model of how mitochondria operate is not universal and that in plants, yeasts and protozoa the core design of mitochondria is modified and modulated in many interesting ways. These adaptations are fascinating to study in their own right and it is important not to become too ‘mammaliocentric’ in considering how mitochondria operate. One illustration of this is the fascinating ways in which non-mammalian enzymes can be introduced into mammalian mitochondria to adapt their metabolism in interesting ways. For example, the introduction of NADH dehydrogenase from yeasts to modulate mitochondria and thereby bypass defects in complex I is now widely used [29]. Another example is the introduction of the alternative oxidase from other organisms into mammalian systems in order to selectively oxidize the CoQ pool [30]. Finally, there is the possibility that the ‘atypical’ mitochondrial processes found in protazoan parasites such as trypanosomes or plasmodia may lead to new drugs that affect the parasite, but not the host [31].

There is a wide range of other disorders in which mitochondrial disruption plays a significant role, including sepsis, neurodegeneration, metabolic syndrome, organ transplantation, cancer, autoimmune diseases and diabetes [1,32]. Consequently, mitochondria are an important node for therapeutic intervention, even if damage to the actual organelle is not the initial pathological event [32,33]. Therefore, it is clear that understanding mitochondria better provides new insights into many different aspects of basic cell physiology. In addition, mitochondrial dysfunction will also contribute to pathologies and thus a better understanding of mitochondria may help us better understanding the nature of these pathologies and thus enable us to develop better therapies and diagnostic methods [1,2,16,34].

## 3. What don't we know about mitochondria?

From the above section, it is clear that there is evidence for multiple central roles of mitochondria in the life and death of the cell. From this extensive list, one might assume that our understanding of mitochondrial function is near completion; however, the truth is there are vast areas of profound ignorance of how mitochondria operate. We do know a lot about the structure of mitochondrial proteins, particularly of the molecular machines that generate and use the protonmotive force to generate ATP [35]. We are also learning a lot more about how the mitochondrial genome is expressed, about the structure of the mitochondrial inner membrane and how mitochondrial dynamics integrates the organelle into the rest of the cell.

However, despite these advances, there are still huge gaps in our knowledge. In our view, one of the most intriguing of these challenges is to understand how mitochondrial functions are integrated into those of the rest of the cell and how these adaptations and communications alter and respond on timescales from seconds to years. For example, we know little about how the expression of the nuclear and mitochondrial genomes is integrated to allow cells to adapt to environmental and developmental challenges. Another major area is how mitochondrial redox signals and the redistribution of metabolites from mitochondria to the cytosol feedback to allow the integration of the organelle into the cell by modulating signalling pathways and gene expression. There are many other similar processes in which mitochondrial function is embedded within that of the cell and continually feeds back on both short and long timescales to integrate mitochondrial function into that of the cell. However, our knowledge of these processes and the web of interactions that link mitochondrial function to the rest of the cell is something we are only just starting to learn about.

## 4. Why use chemical biology to study mitochondria?

Of course, a huge range of investigative techniques can and should be applied to the study of mitochondrial function. Our view is that the application of chemical biology has the potential to make significant contributions to addressing many of critical uncertainties facing us now in mitochondrial studies. Namely, how do mitochondria function in the cell and *in vivo* and how does this integration adapt over time to short-term and long-term environmental challenges and to development, pathology and ageing? In this the versatility of biological chemistry enables us to develop tools that report on aspects of mitochondria and also to manipulate mitochondrial function. Specifically, the use of tools to modify and report on small molecules and to alter proteins and nucleic acids has the potential to provide insights into function. In particular, chemical biology has the potential to use structure–function relationships in probing the biochemistry of the organelle and also to develop new therapeutics. In addition, these can be addressed by the development of drugs to selectively alter mitochondrial function in pathology.

## 5. Current strategies for using chemical biology to assess and intervene in mitochondrial function

**Figure 2.**
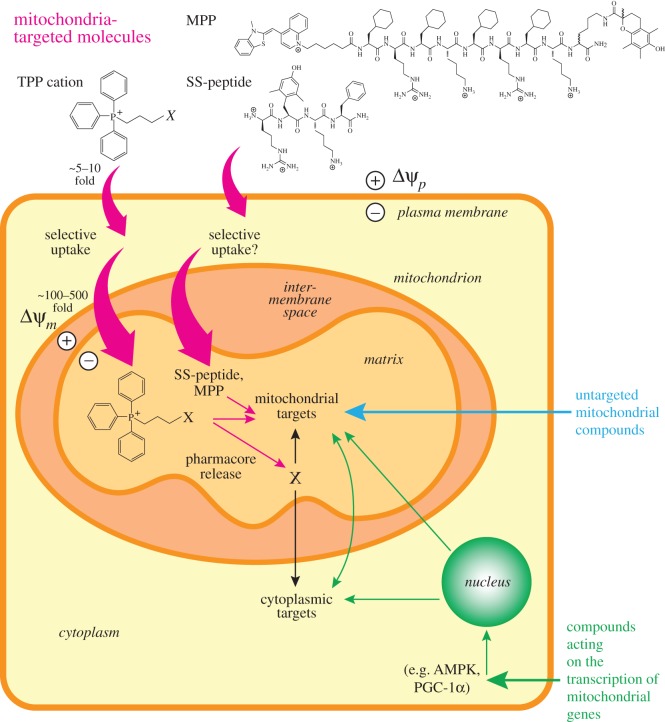
General strategies to use small molecules to affect mitochondria. The first approach is by targeting compounds to mitochondria. This can be done by conjugation to a lipophilic cation such as triphenylphosphonium (TPP), which can lead to the selective uptake of the attached bioactive moiety or pharmacophore (X) into the mitochondrial matrix. Alternatively, peptides that accumulate within mitochondria such as the SS or MPP peptides can be used. All these mitochondria-targeted compounds can act within the mitochondria, or the active pharmacophore can be released from the targeting moiety within the mitochondrion. In addition, compounds that are not targeted to mitochondria but which act there by binding to specific targets can be used. Finally, many compounds can influence mitochondria by affecting processes outside mitochondria, such as the activity of kinases, transcription factors, or transcriptional coactivators.

While the application of chemical biology to mitochondria has only been going for a decade or so, a number of general strategies have emerged. For example, three broad approaches have been used to assess and intervene in mitochondria (figure 2): either designing molecules that are targeted selectively to mitochondria in order to deliver fluorophores, probes, sensors or inhibitors, those that acts on factors (e.g. transcription factors) that alter mitochondria, or to make molecules that are untargeted but which only affect the mitochondrion. These approaches have been extensively reviewed over the past few years [3,5,34,36–38], so here we just outline these approaches. Targeting of large molecules such as proteins, to mitochondria has been achieved by the use of mitochondrial import sequences. The other approaches to target small molecules to mitochondria have focused on the use of lipophilic cations such as TPP and also peptides that are taken up by mitochondria [5,34]. These have been used to send probe molecules to mitochondria. A second approach has been to target transcription factors or other signalling pathways that will alter mitochondrial function [39]. The final class of molecule has been those that are not targeted but which respond to targets in mitochondria, such as uncouplers or those that act on cyclophilin D [40]. All of these approaches are now being increasingly widely used to assess and intervene in mitochondrial function and in the development of drugs.

## 6. Current challenges in applying chemical biology to mitochondria

While the field of applying chemical biology to mitochondria has made significant progress, there are very significant challenges remaining that, if addressed, would greatly enhance the utility of the approaches. Here, we outline some of the major challenges and also discuss how it may be possible to address some of them.

**Figure 3.**
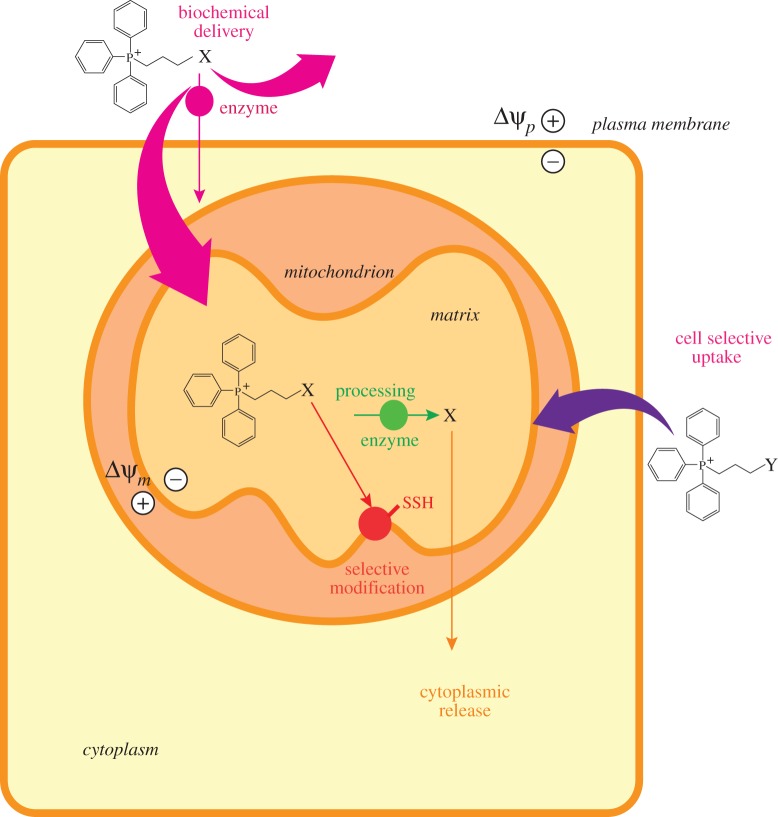
Potential strategies for the selective delivery of mitochondria-targeted compounds to mitochondria *in vivo*. Here, two mitochondria targeted compounds, X and Y, are illustrated. In one of them, X, the compound is normally excluded from cells but is processed by an enzyme on the surface of this cell type, to locally enable the compound to be taken up into the cell and thus to mitochondria in only one cell type. For the compound Y an alternative strategy is used, in which the compound is selectively taken up by a particular cell and once there is further accumulated into the mitochondria.

While the use of lipophilic cations and targeting peptides can deliver drugs and probes to mitochondria *in vivo*, the selective delivery to mitochondria within different organs is not possible. There is preferential uptake by some organs, such as the heart, but this depends on a number of factors including site and mode of delivery of the compound and the local membrane potentials and vascularization of the organs. A general strategy for organ selective delivery, or to particular cells types such as cancer cells, would be of great benefit. Ways that this might be approached are by modular approaches, with a unit designed to deliver the compound to the cell or tissue type, followed by its release there and mitochondrial uptake (figure 3). Alternatively, a mirror image of this modular approach could be considered in which the compound is excluded from all but the target cell or tissue, for example, by the removal of a factor excluding the compound by an enzyme on the surface of the target cells (figure 3).

A further challenge is to develop methods for the delivery of large molecules to mitochondria *in vivo*. While the ectopic delivery of proteins can be routinely achieved by conjugation to a mitochondrial import sequence in conjunction with appropriate expression systems, the delivery of other large molecules such as nucleic acids or their analogues has been difficult. Current approaches using the protein import machinery seem promising. However, other modes such as the use of nanoparticles, or liposomes have yet to show convincing evidence of delivery.

A final challenge is the ability to assess mitochondria *in vivo* in real time. This can be done in cells in culture by use of a range of fluorescent probes, but *in vivo* this is only possible at the surfaces of tissues or in small, transparent organisms such as zebra fish embryos. The ability to monitor mitochondria in tissues *in vivo* in large organisms or in patients would be very useful. While the use of mitochondria-targeted probes assessed by mass spectrometry has proven useful [41], this usually requires the extraction of tissues for analysis, which complicates the ability to infer what happens *in vivo*. The analysis of such compounds in urine may enable a less invasive measurement [42], but this approach is still in its infancy. Perhaps more promising is the possibility of combining mitochondria targeting and assessment with methods that enable visualization in the whole organism in real time, for example, the use of PET imaging has shown promise, but it may be possible to extend this to other methods such as MRS using hyperpolarization.

## 7. Conclusion

The use of chemical biology approaches to manipulate and report on mitochondria is in its infancy. While there have been considerable developments, this interdisciplinary field is really just starting and the expectation is that the continued interactions and iterations between chemists and a range of biological and medical scientists will lead to many new insights into how mitochondria operate in health and disease as well as generating new therapeutic possibilities.

## References

[1] Wallace DC, Fan W, Procaccio V 2010 Mitochondrial energetics and therapeutics. Annu. Rev. Pathol. 5, 297–348. (10.1146/annurev.pathol.4.110807.092314) 20078222 PMC3245719

[2] Duchen MR, Szabadkai G 2010 Roles of mitochondria in human disease. Essays Biochem. 47, 115–137. (10.1042/bse0470115) 20533904

[3] Smith RA, Hartley RC, Cocheme HM, Murphy MP 2012 Mitochondrial pharmacology. Trends Pharmacol. Sci. 33, 341–352. (10.1016/j.tips.2012.03.010) 22521106

[4] Murphy E, Bers D, Rizzuto R 2009 Mitochondria: from basic biology to cardiovascular disease. J. Mol. Cell. Cardiol. 46, 765–766. (10.1016/j.yjmcc.2009.03.004) 19289126 PMC2700026

[5] Smith RA, Hartley RC, Murphy MP 2011 Mitochondria-targeted small molecule therapeutics and probes. Antiox Redox Signal. 15, 3021–3038. (10.1089/ars.2011.3969) 21395490

[6] Paul VD, Lill R 2015 Biogenesis of cytosolic and nuclear iron-sulfur proteins and their role in genome stability. Biochim. Biophys. Acta. 1853, 1528–1539. (10.1016/j.bbamcr.2014.12.018) 25583461

[7] Ye H, Rouault TA 2010 Human iron-sulfur cluster assembly, cellular iron homeostasis, and disease. Biochemistry 49, 4945–4956. (10.1021/bi1004798) 20481466 PMC2885827

[8] Wallace DC 2012 Mitochondria and cancer. Nat. Rev. Cancer. 12, 685–698. (10.1038/nrc3365) 23001348 PMC4371788

[9] Chouchani ET, Pell VR, James AM, Work LM, Saeb-Parsy K, Frezza C, Krieg T, Murphy MP 2016 A unifying mechanism for mitochondrial superoxide production during ischemia–reperfusion injury. Cell Metab. 23, 254–263. (10.1016/j.cmet.2015.12.009) 26777689

[10] Sciacovelli M et al. 2016 Fumarate is an epigenetic modifier that elicits epithelial-to-mesenchymal transition. Nature 537, 544–547. (10.1038/nature19353) 27580029 PMC5136292

[11] Murley A, Nunnari J 2016 The emerging network of mitochondria–organelle contacts. Mol. Cell. 61, 648–653. (10.1016/j.molcel.2016.01.031) 26942669 PMC5554544

[12] Mammucari C, Patron M, Granatiero V, Rizzuto R 2011 Molecules and roles of mitochondrial calcium signaling. Biofactors 37, 219–227. (10.1002/biof.160) 21674643

[13] Brand MD, Murphy MP 1987 Control of electron flux through the respiratory chain in mitochondria and cells. Biol. Rev. 62, 141–193. (10.1111/j.1469-185X.1987.tb01265.x) 3300795

[14] Wai T et al. 2016 The membrane scaffold SLP2 anchors a proteolytic hub in mitochondria containing PARL and the i-AAA protease YME1 L. EMBO Rep. 17, 1844–1856. (10.15252/embr.201642698) 27737933 PMC5283581

[15] Tait SW, Green DR 2010 Mitochondria and cell death: outer membrane permeabilization and beyond. Nat. Rev. Mol. Cell Biol. 11, 621–632. (10.1038/nrm2952) 20683470

[16] Murphy E, Steenbergen C 2011 What makes the mitochondria a killer? Can we condition them to be less destructive? Biochim. Biophys. Acta. 1813, 1302–1308. (10.1016/j.bbamcr.2010.09.003) 20837069 PMC3398608

[17] Halestrap A 2005 Biochemistry: a pore way to die. Nature 434, 578–579. (10.1038/434578a) 15800609

[18] Rasola A, Bernardi P 2011 Mitochondrial permeability transition in Ca^2+^-dependent apoptosis and necrosis. Cell Calcium 50, 222–233. (10.1016/j.ceca.2011.04.007) 21601280

[19] Chouchani ET et al. 2016 Mitochondrial ROS regulate thermogenic energy expenditure and sulfenylation of UCP1. Nature 532, 112–116. (10.1038/nature17399) 27027295 PMC5549630

[20] Brand MD 1990 The contribution of the leak of protons across the mitochondrial inner membrane to standard metabolic rate. J. Theoret. Biol. 145, 267–286. (10.1016/S0022-5193(05)80131-6) 2169556

[21] Murphy MP 2009 How mitochondria produce reactive oxygen species. Biochem. J. 417, 1–13. (10.1042/BJ20081386) 19061483 PMC2605959

[22] Arnoult D, Soares F, Tattoli I, Girardin SE 2011 Mitochondria in innate immunity. EMBO Rep. 12, 901–910. (10.1038/embor.2011.157) 21799518 PMC3166463

[23] Tormos KV, Anso E, Hamanaka RB, Eisenbart J, Joseph J, Kalyanaraman B, Chandel NS 2011 Mitochondrial complex III ROS regulate adipocyte differentiation. Cell Metab. 14, 537–544. (10.1016/j.cmet.2011.08.007) 21982713 PMC3190168

[24] Holmstrom KM, Finkel T 2014 Cellular mechanisms and physiological consequences of redox-dependent signalling. Nat. Rev. Mol. Cell Biol. 15, 411–421. (10.1038/nrm3801) 24854789

[25] Green DR, Galluzzi L, Kroemer G 2011 Mitochondria and the autophagy–inflammation–cell death axis in organismal aging. Science 333, 1109–1112. (10.1126/science.1201940) 21868666 PMC3405151

[26] Zhou R, Yazdi AS, Menu P, Tschopp J 2011 A role for mitochondria in NLRP3 inflammasome activation. Nature 469, 221–225. (10.1038/nature09663) 21124315

[27] Buskiewicz IA et al. 2016 Reactive oxygen species induce virus-independent MAVS oligomerization in systemic lupus erythematosus. Sci Signal. 9, ra115. (10.1126/scisignal.aaf1933) PMC5321043 27899525

[28] Hanahan D, Weinberg RA 2011 Hallmarks of cancer: the next generation. Cell 144, 646–674. (10.1016/j.cell.2011.02.013) 21376230

[29] Sanz A et al. 2010 Expression of the yeast NADH dehydrogenase Ndi1 in *Drosophila* confers increased lifespan independently of dietary restriction. Proc. Natl Acad. Sci. USA 107, 9105–9110. (10.1073/pnas.0911539107) 20435911 PMC2889079

[30] Fernandez-Ayala DJ et al. 2009 Expression of the *Ciona intestinalis* alternative oxidase (AOX) in *Drosophila* complements defects in mitochondrial oxidative phosphorylation. Cell Met. 9, 449–460. (10.1016/j.cmet.2009.03.004) 19416715

[31] Dunn EA, Roxburgh M, Larsen L, Smith RAJ, McLellan AD, Heikal A, Murphy MP, Cook GM 2014 Incorporation of triphenylphosphonium functionality improves the inhibitory properties of phenothiazine derivatives in *Mycobacterium tuberculosis.* Bioorg. Med. Chem. 22, 5320–5328. (10.1016/j.bmc.2014.07.050) 25150092

[32] Murphy MP 2009 Mitochondria—a neglected drug target. Curr. Opin Investig. Drugs 10, 1022–1024. 19777389

[33] Balaban RS, Nemoto S, Finkel T 2005 Mitochondria, oxidants, and aging. Cell 120, 483–495. (10.1016/j.cell.2005.02.001) 15734681

[34] Murphy MP, Smith RA 2007 Targeting antioxidants to mitochondria by conjugation to lipophilic cations. Ann Rev Pharmacol Toxicol. 47, 629–656. (10.1146/annurev.pharmtox.47.120505.105110) 17014364

[35] Nicholls DG, Ferguson SJ 2013 Bioenergetics 4. London, UK: Academic Press.

[36] Szeto HH, Schiller PW 2011 Novel therapies targeting inner mitochondrial membrane—from discovery to clinical development. Pharmaceut. Res. 28, 2669–2679. (10.1007/s11095-011-0476-8) 21638136

[37] Yousif LF, Stewart KM, Kelley SO 2009 Targeting mitochondria with organelle-specific compounds: strategies and applications. Chembiochem. 10, 1939–1950. (10.1002/cbic.200900185) 19637148

[38] Jean SR, Ahmed M, Lei EK, Wisnovsky SP, Kelley SO 2016 Peptide-mediated delivery of chemical probes and therapeutics to mitochondria. Accs. Chemical Res. 49, 1893–1902. (10.1021/acs.accounts.6b00277) 27529125

[39] Viscomi C, Bottani E, Civiletto G, Cerutti R, Moggio M, Fagiolari G, Schon EA, Lamperti C, Zeviani M 2011 *In vivo* correction of COX deficiency by activation of the AMPK/PGC-1alpha axis. Cell Metab. 14, 80–90. (10.1016/j.cmet.2011.04.011) 21723506 PMC3130927

[40] Piot C et al. 2008 Effect of cyclosporine on reperfusion injury in acute myocardial infarction. N Engl. J. Med. 359, 473–481. (10.1056/NEJMoa071142) 18669426

[41] Logan A et al. 2014 Using exomarkers to assess mitochondrial reactive species *in vivo*. Biochim. Biophys. Acta. 1840, 923–930. (10.1016/j.bbagen.2013.05.026) 23726990

[42] Pun PB et al. 2014 A mitochondria-targeted mass spectrometry probe to detect glyoxals: implications for diabetes. Free Rad. Biol. Med. 67, 437–450. (10.1016/j.freeradbiomed.2013.11.025) 24316194 PMC3978666

